# Empirical method for rapid quantification of intrinsic fluorescence signals of key metabolic probes from optical spectra measured on tissue-mimicking turbid medium

**DOI:** 10.1117/1.JBO.26.4.045001

**Published:** 2021-04-23

**Authors:** Tengfei Sun, Caigang Zhu

**Affiliations:** University of Kentucky, Department of Biomedical Engineering, Lexington, Kentucky, United States

**Keywords:** fluorescence, spectroscopy, attenuation correction, absorption, scattering

## Abstract

**Significance:** Optical fluorescence spectroscopy technique has been explored extensively to quantify both glucose uptake and mitochondrial metabolism with proper fluorescent probes in small tumor models *in vivo*. However, it remains a great challenge to rapidly quantify the intrinsic metabolic fluorophores from the optically measured fluorescence spectra that contain significant distortions due to tissue absorption and scattering.

**Aim:** To enable rapid spectral data processing and quantify the *in vivo* metabolic parameters in real-time, we present an empirical ratio-metric method for rapid fluorescence spectra attenuation correction with high accuracy.

**Approach:** A first-order approximation of intrinsic fluorescence spectra can be obtained by dividing the fluorescence spectra by diffuse reflectance spectra with some variable powers. We further developed this approximation for rapid extraction of intrinsic key metabolic probes (2-NBDG for glucose uptake and TMRE for mitochondrial function) by dividing the distorted fluorescence spectra by diffuse reflectance intensities recorded at excitation and emission peak with a pair of system-dependent powers. Tissue-mimicking phantom studies were conducted to evaluate the method.

**Results:** The tissue-mimicking phantom studies demonstrated that our empirical method could quantify the key intrinsic metabolic probes in near real-time with an average percent error of ∼5%.

**Conclusions:** An empirical method was demonstrated for rapid quantification of key metabolic probes from fluorescence spectra measured on a tissue-mimicking turbid medium. The proposed method will potentially facilitate real-time monitoring of key metabolic parameters of tumor models *in vivo* using optical spectroscopy, which will significantly advance translational cancer research.

## 1 Introduction

Both glycolysis and mitochondrial metabolism play a key role in understanding how the metabolic characteristics of tumors impact therapeutic outcome and can be used in a wide range of cancer pharmacology applications to improve the effectiveness of cancer therapy.^1^ Some tumors can switch their metabolism between glycolysis and mitochondrial metabolism to survive under hostile conditions and therapeutic stress.^2^ These “adaptable” tumors with a capacity to rely on both glycolytic and mitochondrial metabolism promote negative outcomes, such as increased recurrence,^3^ migration,^4^ and metastatic propensity.^5^ Therefore, it is crucial to be able to track glycolysis, mitochondrial metabolism, and their interplay in tumors to combat this intractable disease.

Several tools with a variety of length scales have been developed to measure the metabolic parameters in cancer cells or *in vivo* tumors for cancer research. The Seahorse assay is frequently used to measure mitochondrial respiration and glycolysis of *in vitro* cells by capturing the oxygen consumption rate and extracellular acidification rates.^6^ Metabolomics is widely used to simultaneously screen a large number of metabolites and map metabolic networks from *in vitro* cells to *ex vivo* tissues.^7^ Positron emission tomography is used to image glucose uptake *in vivo* at the organ level.^8^ Magnetic resonance spectral imaging is used to report tumor mitochondrial metabolism and glycolysis via P31 or C13 *in vivo*.^9^^,^^10^ Biomedical optical spectroscopy techniques have gained increasing interest in measuring glycolysis and mitochondrial function of tumors *in vivo* for cancer research due to their cost-effectiveness and high portability.^11^^–^^14^ Our group has developed techniques to quantify glucose uptake using glucose analog 2-NBDG^15^ and mitochondrial membrane potential using TMRE^16^ in tumor models *in vivo*.^11^^,^^12^

A significant challenge for using biomedical optical spectroscopy to quantify metabolic parameters *in vivo* is to effectively remove the attenuation caused by tissue absorption and scattering on the measured fluorescence signals.^17^ We previously developed an inverse scalable Monte Carlo-based model to extract intrinsic fluorescence from turbid media with high accuracy and demonstrated this technique for quantification of 2-NBDG and TMRE uptake on small tumor models *in vivo*.^11^^,^^12^ However, it is difficult to adapt the Monte Carlo technique for real-time data processing due to the time-consuming fitting processing.^13^ To enable real-time spectral data processing, we have developed and now present an empirical ratiometric method for rapid fluorescence spectra attenuation correction. Specifically, we report an empirical technique for rapid extraction of intrinsic 2-NBDG and TMRE fluorescence by dividing the measured fluorescence spectra by diffuse reflectance intensities recorded at excitation and emission peak with a pair of system-dependent powers. To demonstrate the proof-of-concept of our empirical technique, we performed tissue-mimicking turbid fluorescence phantom studies using a custom-designed optical spectroscopy platform. Our phantom study data show that the average percent error for extraction of both 2-NBDG and TMRE fluorophores decreases from 80% to 5% with the use of our empirical method. The method will potentially facilitate real-time monitoring of key metabolic parameters of tumor models *in vivo* using optical spectroscopy techniques, which will significantly advance translational cancer research.

## 2 Materials and Method

### 2.1 Empirical Ratiometric Model

A first-order approximation of intrinsic fluorescence spectra can be obtained by dividing the fluorescence spectra by diffuse reflectance spectra recorded at the same anatomical location to some variable power.^18^ This approximation can be expanded to remove the distortions by dividing the fluorescence spectra by integrated diffuse reflectance spectra recorded at both absorption band and emission band with a variable power, which has been used for rapid extraction of pPIX fluorophore.^19^ We further developed this technique for rapid extraction of intrinsic 2-NBDG and TMRE fluorescence by dividing the fluorescence spectra by reflectance intensities recorded at excitation and emission peak with a pair of system-dependent power, using the following equation:

$$F_{\mathrm{corr}}(\lambda )=\frac{F_{\text{raw}}(\lambda )}{{\left[R_{\mathrm{ex}}\right]}^{\alpha }{\left[R_{\mathrm{em}}\right]}^{\beta }},$$ (1)

$F_{\mathrm{corr}}(\lambda )$ represents the intrinsic fluorescence spectrum, and $F_{\text{raw}}(\lambda )$ denotes the raw fluorescence spectrum that contains distortions caused by hemoglobin absorption and tissue scattering. $R_{\mathrm{ex}}$ and $R_{\mathrm{em}}$ represent the diffuse reflectance intensities at excitation and emission region, respectively, which can be either the diffuse reflectance at a single wavelength such as $R_{\mathrm{ex}}=R(\lambda_{\mathrm{ex}})$ or the summed diffuse reflectance intensities within a chosen wavelength band such as $R_{\mathrm{ex}}=\overset{\lambda_{\mathrm{ex}2}}{\underset{\lambda_{\mathrm{ex}1}}{\sum }}R(\lambda )$. The parameters α and β are the powers that indicate the extent of modulation caused by tissue absorption and scattering.

### 2.2 Tissue-Mimicking Phantom Experiment

We created tissue-mimicking fluorescence phantoms to evaluate the empirical method for rapid quantification of metabolic probes. 20% intralipid (Sigma-Aldrich) was used to mimic tissue scattering. Dehydrated human hemoglobin powder (H0267, Sigma-Aldrich) was used as a major absorber in the phantoms. TMRE and 2-NBDG were used as fluorophores. Phosphate buffered saline 1× (Fisher Scientific) was used to suspend the scattering intralipid, hemoglobin, and fluorophores for the liquid fluorescence phantoms. The optical properties of the phantoms were designed based on the formerly reported optical properties of mice skin.^20^ Specifically, the tissue-mimicking phantoms had the following average absorption coefficients and reduced scattering coefficients (400 to 600 nm): $\mu_{a}=[1.5,3.0,4.5]\;{\mathrm{cm}}^{-1}$ and $\mu_{s}^{\prime }=[4.5,9.0,13.5]\;{\mathrm{cm}}^{-1}$. For every single set of absorption and scattering phantom, either 2-NBDG or TMRE was added at biologically relevant concentrations to mimic the fluorophore uptake in tissues. Specifically, the 2-NBDG was varied from 0 to 9  μM by every 3  μM, and the TMRE was varied from 0 to 300 nM by every 100 nM based on our former *in vivo* studies.^11^ All baseline absorption and scattering phantoms (no fluorophore) had the same starting volume of 5 mL, then a stock of 15  μL 2-NBDG or TMRE was added sequentially to create the fluorescence phantoms with the designed fluorophore concentrations. Each phantom was vibrated well to ensure homogeneity before the optical measurements. Totally, 27 sets of fluorescence phantoms were made for each fluorescent probe. Independently, two sets of liquid phantoms consisting of either 2-NBDG or TMRE only but with the same set of concentrations were made and used as control references for future comparison.

### 2.3 Spectroscopy System and Optical Measurement

A portable spectroscopy system based on a Solis™ white LED (400 to 900 nm, SOLIS-3C, Thorlabs) and a compact visible spectrometer (FLAME-T-VIS-NIR, Ocean Optics) was used for diffuse reflectance and fluorescence measurements. A low-cost fiber probe (BF19Y2HS02, Thorlabs) with 10 illumination fibers and 9 collection fibers was used for light delivery and collection. Two bandpass filters were used in the illumination channel to generate appropriate excitations for 2-NBDG and TMRE. Specifically, a bandpass filter of 450 nm (25 nm bandwidth) was used to excite 2-NBDG as its excitation peak is around 470 nm, whereas a bandpass filter of 549 nm (25 nm bandwidth) was used to excite TMRE because its excitation peak is around 550 nm. In the collection end, two long-pass filters (515 nm for 2-NBDG, and 575 nm for TMRE) were used for fluorescence measurement. No filter was used in the collection channel for diffuse reflectance measurement thus diffuse reflectance in a wide range (400 to 900 nm) was measured for future correction; however, one neutral density filter (OD=2.0) was installed on the diffuse reflectance illumination channel to protect the spectrometer from overexposure. Two cage filter wheels (CFW6, Thorlabs) were installed on the excitation branch and emission branch, allowing a fast switch among three measurement channels: diffuse reflectance, 2-NBDG fluorescence, and TMRE fluorescence. Both diffuse reflectance and fluorescence spectra were measured on every single set of fluorescence phantom by loading the fiber probe into the middle level of a phantom. The integration time was set as 50 ms for diffuse reflectance and 5 s for fluorescence measurement. After all optical measurements on the phantoms were completed, reference spectra on a diffuse reflectance standard puck (20%, Spectralon, Labsphere) and a fluorescence standard puck (USF210-010, LabSphere) were collected for future calibration.

### 2.4 Data Analysis

All diffuse reflectance and fluorescence spectra were calibrated using a 20% reflectance standard (Spectralon, Labsphere) and a fluorescence standard (USF 210-010, LabSphere), respectively. Specifically, the diffuse reflectance spectra measured on samples were calibrated for the wavelength-dependent response by normalizing them to the diffuse reflectance spectrum measured on the reflectance standard. The fluorescence spectral intensities were normalized to the output excitation power quantified by the fluorescence standard, to account for the wavelength-dependent variation of the excitation light intensity. The empirical ratiometric model was implemented in MATLAB (MathWorks, Natick, Massachusetts, USA) for spectral data processing. The optimal parameter set for the pair of powers [α,β] that could converge the fluorescence spectra from different absorption–scattering combinations but the same fluorophore concentration was found for the selected correction wavelengths or wavelength bands. Specifically, the relative variation of corrected fluorescence in the selected group of phantoms with the same fluorophore concentrations was minimized using the empirical model. The nonlinear multivariable optimization function, fmincon from the MATLAB optimization toolbox, was used to find the optimal parameter set [α,β]. Once the optimal parameter set [α,β] was found from the selected group of phantoms (15 from 27 phantoms), the whole set of fluorescence spectral data (15 sets of training phantoms plus 12 sets of validation phantoms) was processed and compared with the control references obtained from the control phantoms. The mean percent errors were calculated for the comparison between the corrected fluorescence $F_{\mathrm{corr}}$ with reference set. We further estimated the concentrations of the metabolic probes based on the corrected fluorescence spectra intensities at their corresponding emission peaks. The estimated concentrations were then compared with their corresponding true values using Pearson’s test.

## 3 Results

### 3.1 Attenuation Correction for 2-NBDG Fluorescence

**Fig. 1:**
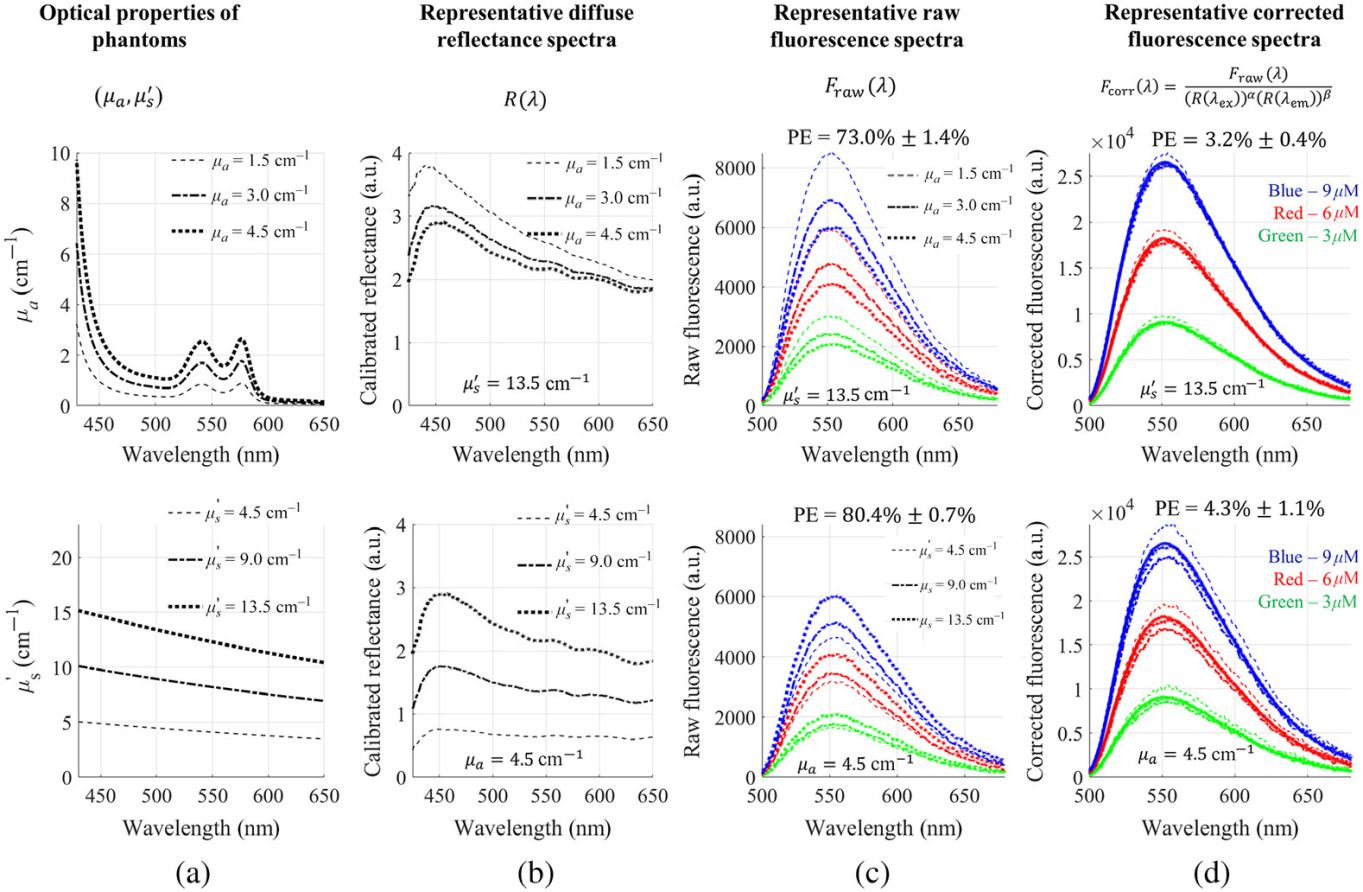
The empirical model with a pair of single wavelengths for accurate attenuation removal in 2-NBDG phantoms. $\lambda_{\mathrm{ex}}=450\;\mathrm{nm}$, $\lambda_{\mathrm{em}}=550\;\mathrm{nm}$, the parameter set [α,β]=[5.96,−6.44]. (a) Optical properties of phantoms; (b) diffuse reflectance spectra; (c) raw fluorescence; and (d) corrected fluorescence of phantoms with fixed reduced scatterings but different absorption coefficients (top) and fixed absorption but different reduced scatterings (bottom).

Figure 1 shows the attenuation correction for 2-NBDG fluorescence using the empirical model. Diffuse reflectance intensities at the excitation source peak (450 nm) and the emission peak (550 nm) with optimized powers were used for the fluorescence correction. Figure 1(a) shows the representative diffuse reflectance spectra for the phantoms with different absorption coefficients (top) and scattering levels (bottom). Figure 1(b) shows the representative raw fluorescence spectra measured on the turbid fluorescence phantoms. Spectra in the same color but different line styles have the same fluorophore concentrations but different absorptions or scattering levels. The spectra with the same line style but different colors had the same absorption-scattering levels but different fluorescent probe concentrations. The graphs in Fig. 1(b) show significant distortions on the fluorescence spectra caused by either absorption or scattering, which were reflected by the huge percent errors. Figure 1(c) shows the representative corrected fluorescence spectra using the empirical model. The comparison between the corrected fluorescence spectra and the true references spectra (solid curves) demonstrates the high accuracy of the empirical model. Specifically, the percent errors were reduced to 3.2% (absorption group) and 4.3% (scattering group). When the model was used to correct the whole set of phantom data, the average percentage error was reduced from 80% to 6%.

**Fig. 2:**
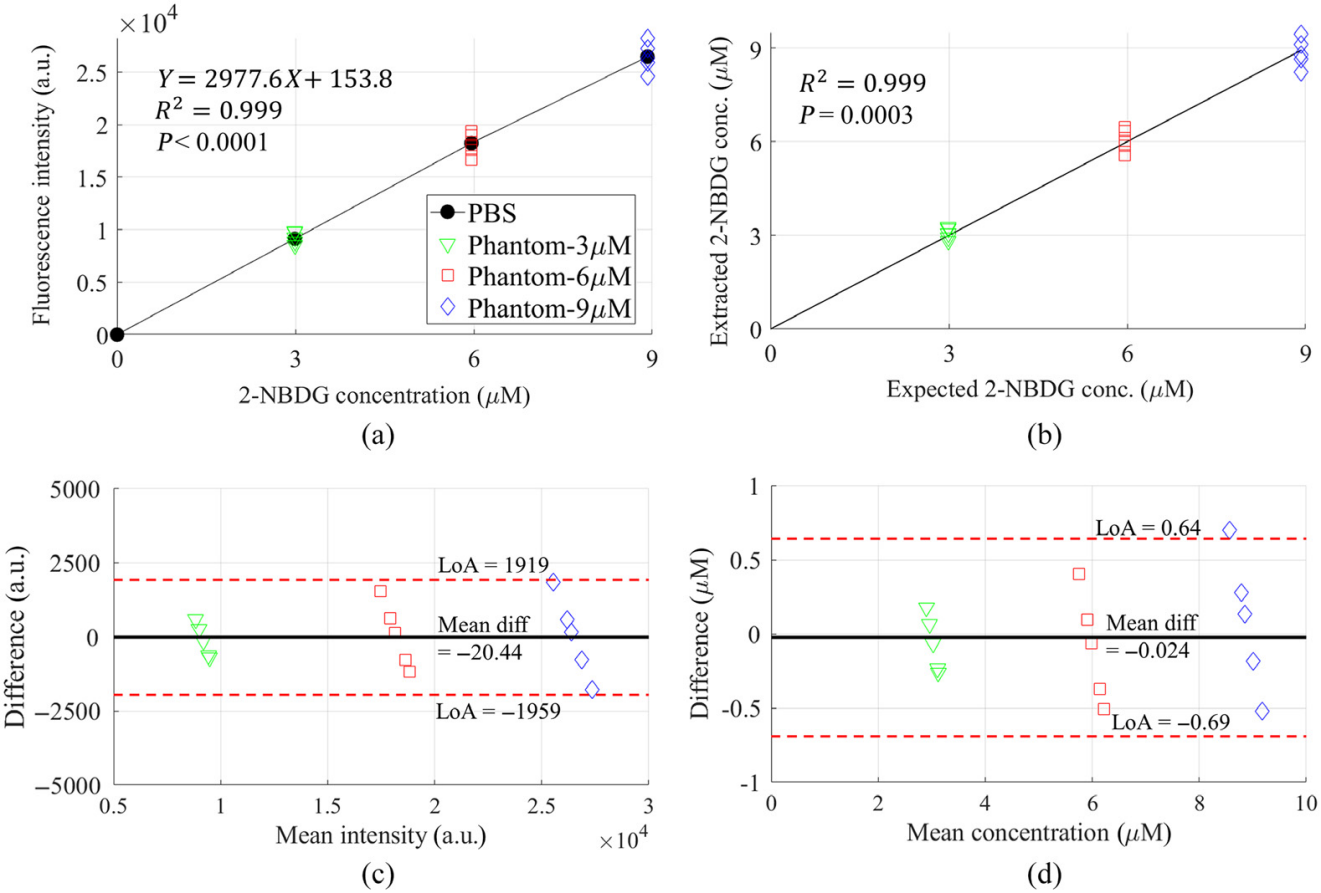
(a) Correlation between the corrected fluorescence intensities at 550 nm and true 2-NBDG concentrations; (b) comparison between the optically 2-NBDG concentrations and their corresponding true concentrations; (c) Bland–Altman plot for data shown in (a); and (d) Bland–Altman plot for (b). LoA represents limits of agreement, which are defined as the mean difference ±1.96  SD of differences. “Mean diff” represents mean difference.

Due to the general interest in metabolic probe concentration quantification in tissue for cancer research, we estimated the 2-NBDG concentrations based on the corrected fluorescence spectra intensities. Figure 2 shows the quantitative comparison between the corrected fluorescence peak intensities and true 2-NBDG concentrations using the processed spectral data. In Fig. 2(a), a linear fit to the corrected fluorescence peak intensities versus 2-NBDG concentrations yielded a coefficient of determination $R^{2}$ of 0.999 and p-value<0.0001, indicating the high performance of the method. Figure 2(b) shows the quantitative comparison between the optically estimated 2-NBDG concentrations with the expected true concentrations. High correlation and accuracy were achieved and demonstrated by the coefficient value and p-value.

**Fig. 3:**
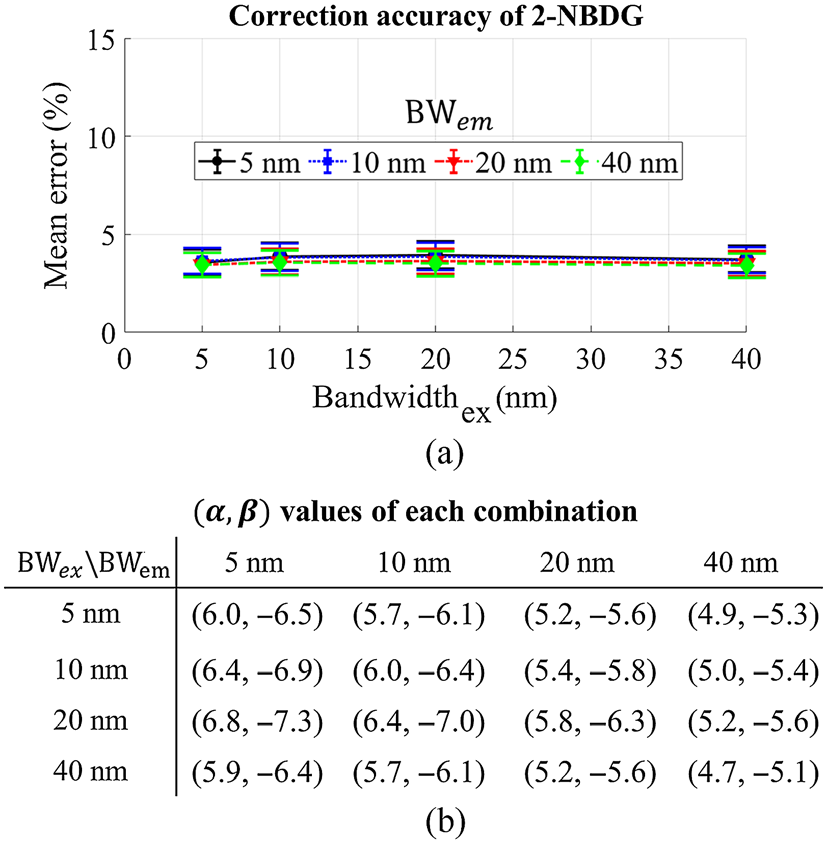
(a) The effect of wavelength bandwidth on the mean percent error of the empirical model for 2-NBDG extraction, and the CWLs were fixed to be 450 and 550 nm. (b) The corresponding parameter set [α,β] for the combination of different wavelength bandwidths.

To further popularize the general use of the empirical model for spectral data processing, we also explored the potentially available wavelength bandwidths and wavelength bands based on the commonly used optical bandpass filters. Figure 3 shows the effect of wavelength bandwidth on the model’s accuracy for 2-NBDG extraction when the central wavelength (CWL) bands for diffuse reflectance were set to be the excitation peak (450 nm) and emission peak (550 nm). Figure 3(a) shows the minimal effect of wavelength bandwidth on performance of the empirical model when the wavelength bands were changed from 5 to 40 nm. However, a larger bandwidth of the optical filters typically would provide enhanced optical signals. Figure 3(b) shows the corresponding powers for each combination of the wavelength bandwidths. Generally, the absolute values of these power decreased slightly when the bandwidths were increased.

**Fig. 4:**
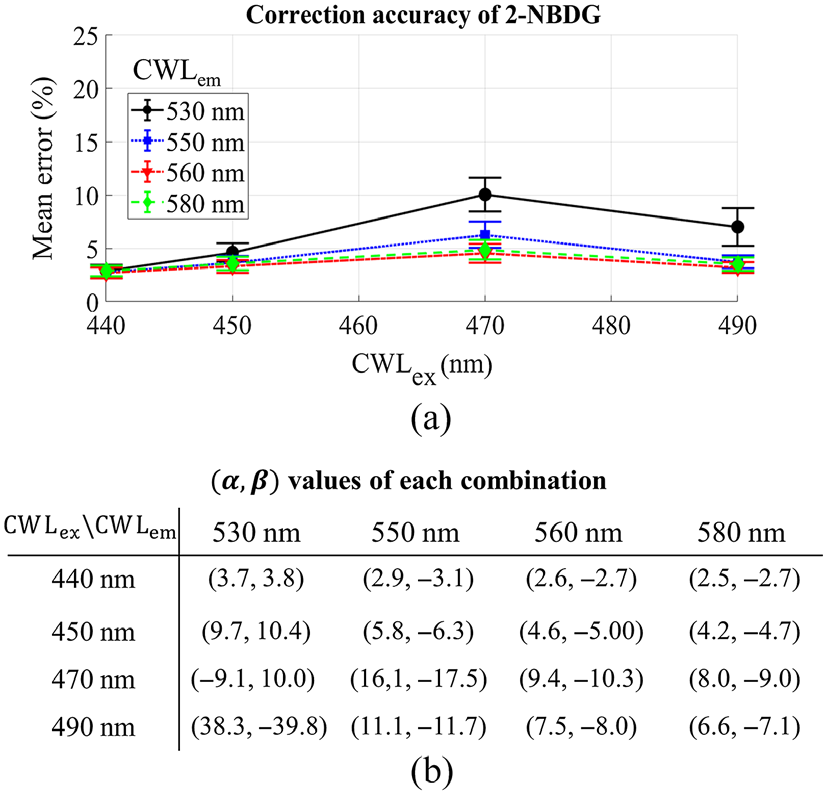
(a) The effect of CWL band of diffuse reflectance collection on the mean percent error of the empirical model for 2-NBDG extraction when bandwidth is fixed at 10 nm. (b) The corresponding powers [α,β] for the combination of different CWLs.

Figure 4 shows the effect of the CWL of diffuse reflectance filters on the accuracy of the empirical model for 2-NBDG correction when the bandwidth of the diffuse reflectance filters is set to 10 nm. The curves with different colors represent the selection of different CWL of filters (${\mathrm{CWL}}_{\mathrm{ex}}$ or ${\mathrm{CWL}}_{\mathrm{ex}}$) for diffuse reflectance collection. Figure 4(a) shows a minimal effect of diffuse reflectance filters’ CWL on the performance of the empirical model when the ${\mathrm{CWL}}_{\mathrm{ex}}$ of diffuse reflectance was set to 440 or 450 nm; however, the accuracy decreases considerably when ${\mathrm{CWL}}_{\mathrm{ex}}$ was changed to 470 and 490 nm, especially when the ${\mathrm{CWL}}_{\mathrm{em}}$ is set to 530 nm.

### 3.2 Attenuation Correction for TMRE Fluorescence

**Fig. 5:**
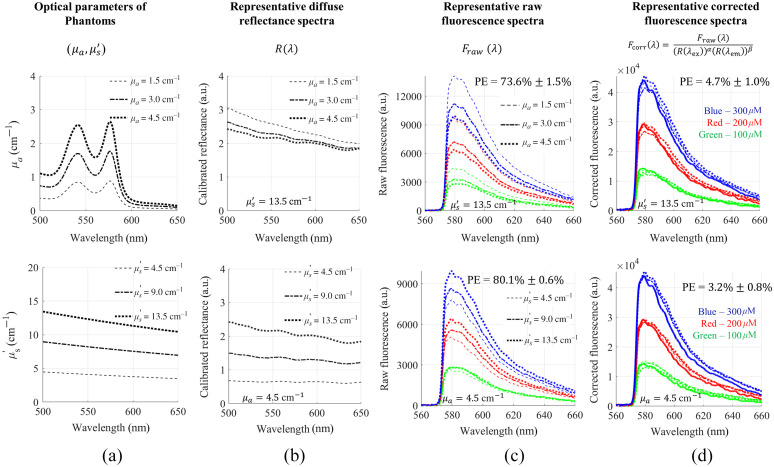
The empirical model with a pair of single wavelengths for accurate attenuation correction in TMRE phantoms. $\lambda_{\mathrm{ex}}=550\;\mathrm{nm}$, $\lambda_{\mathrm{em}}=580\;\mathrm{nm}$, the parameter set [α,β]=[−10.25,10.58]. (a) Optical properties of phantoms; (b) diffuse reflectance spectra; (c) raw fluorescence; and (d) corrected fluorescence of phantoms with fixed reduced scatterings but different absorption coefficients (top) and fixed absorption but different reduced scatterings (bottom).

The attenuation corrections on TMRE spectra are presented in the same manner as the corrections for 2-NBDG spectra. Diffuse reflectance intensities at the excitation peak (550 nm) and the emission peak (580 nm) with an optimized power pair are used for TMRE correction. Similarly, Fig. 5(a) shows the representative diffuse reflectance spectra for the phantoms with different absorption and scattering levels in the range of 520 to 650 nm. The representative raw TMRE fluorescence spectra measured on turbid phantoms in Fig. 5(b) show significant distortions caused by either absorption or scattering, which are reflected by the percent errors of 73.6% and 80.1%, respectively. The comparison between the corrected fluorescence spectra and the true references spectra (solid curves) in Fig. 5(c) show the high performance of the empirical model for distortion removal. The errors were reduced to 4.7% (absorption group) and 3.2% (scattering group), respectively, as shown in Fig. 1(c). To validate the empirical model for TMRE correction, we prepared additional phantoms with lower TMRE concentrations (15 to 50 nM) and absorption or scattering levels at different days. When the same set of powers are used to correct the validation phantom data, an average percent error of 5% for the whole set of data was achieved.

**Fig. 6:**
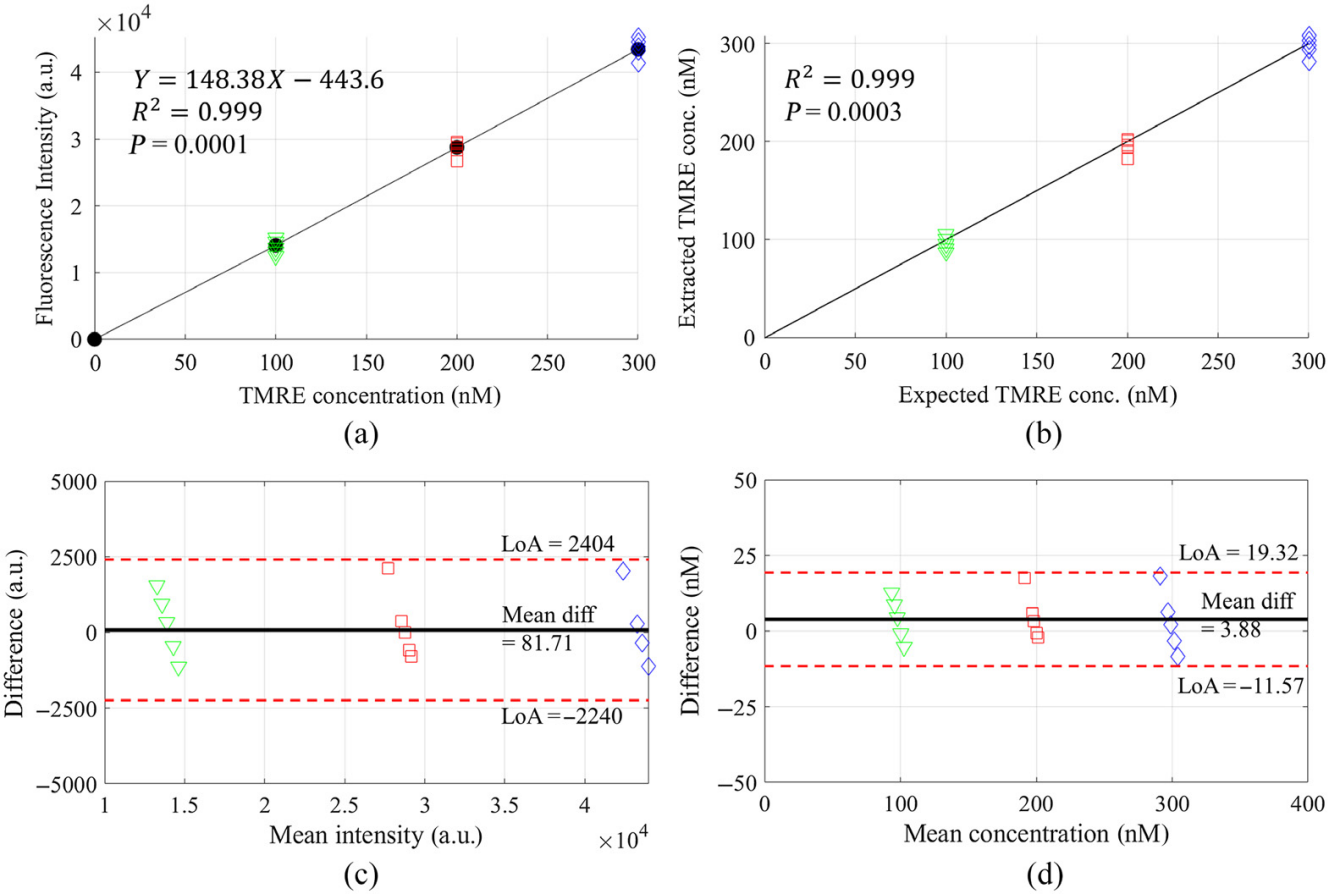
(a) Correlation between the corrected fluorescence intensities at 580 nm and true TMRE concentrations; (b) comparison between the optically TMRE concentrations and their corresponding true concentrations; (c) Bland–Altman plot for data shown in (a); and (d) Bland–Altman plot for (b). LoA represents limits of agreement, which are defined as the mean difference ±1.96  SD of differences. Mean diff represents mean difference.

Figure 6 shows the quantitative comparison between the corrected fluorescence peak intensities and reference TMRE concentrations using the corrected spectra data. In Fig. 6(a), a linear fit to the corrected fluorescence intensities versus TMRE concentrations yields a coefficient of determination $R^{2}$ of 0.999 and p-value<0.0001, indicating the high performance of the empirical correction. Figure 6(b) shows the quantitative comparison between the optically estimated TMRE concentrations with the expected true values. High accuracy is achieved and demonstrated by the low percent error values.

**Fig. 7:**
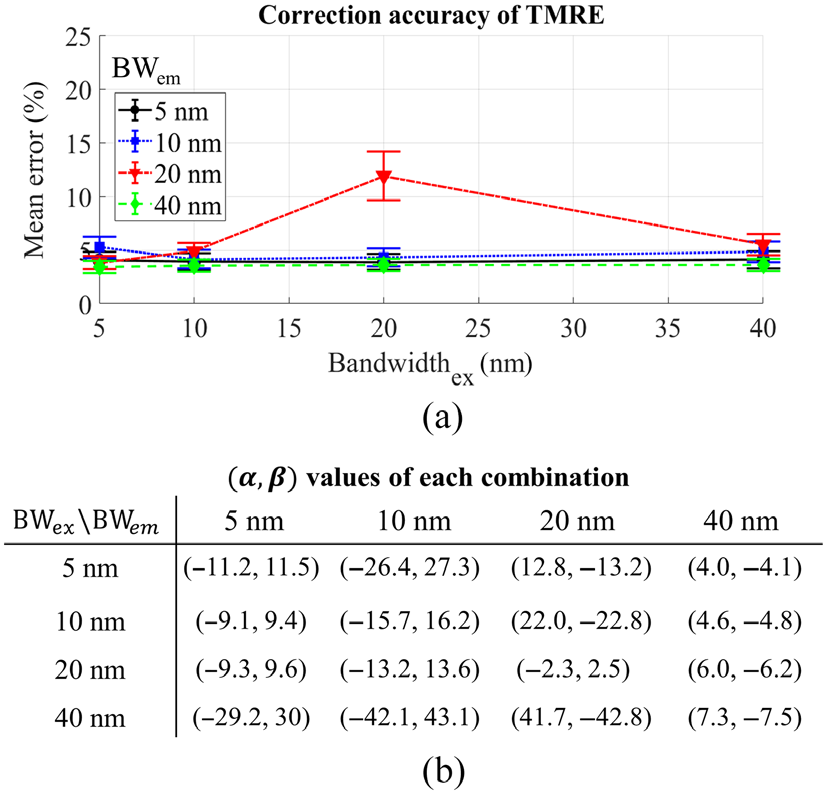
(a) The effect of wavelength bandwidth on the mean percent error of the empirical model for TMRE extraction, the CWLs are fixed at 550 and 580 nm. (b) The corresponding parameter set [α,β] for the combination of different wavelength bandwidths.

Figure 7 shows the effect of wavelength bandwidth on the accuracy of the empirical model for TMRE extraction when the CWL bands for diffuse reflectance are set to the excitation peak (550 nm) and emission peak (580 nm). Figure 7(a) shows a minimal effect of wavelength bandwidth on the performance of the empirical model for TMRE extraction when the wavelength bands are changed from 5 to 40 nm except for the wavelength band of 20 nm. Figure 7(b) shows the corresponding powers for each combination of the wavelength bandwidths. No clear trend is observed on these power values, however, the absolute values of each set of the power α and β were close to each for all combinations as observed in Fig. 3.

**Fig. 8:**
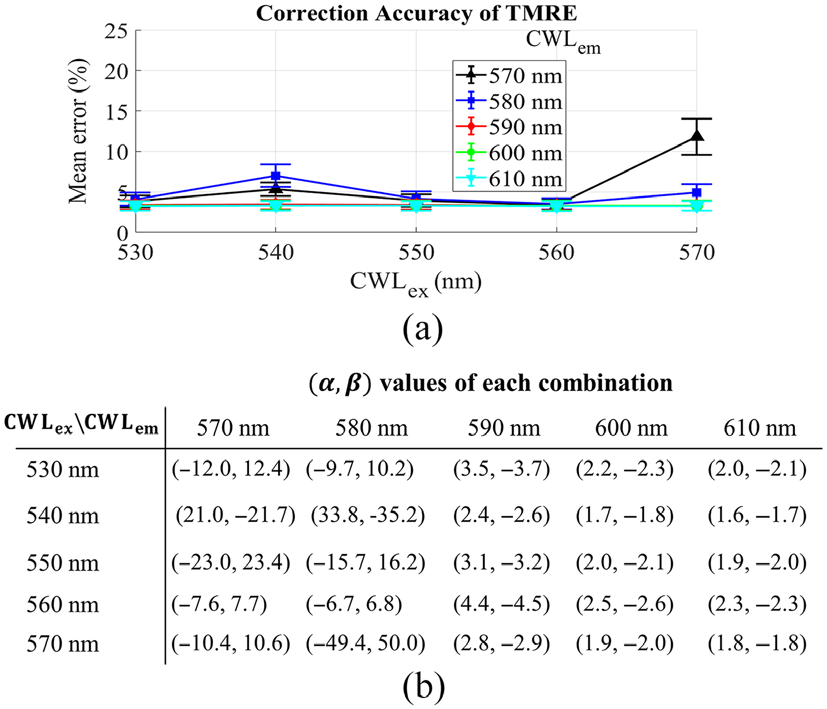
(a) The effect of CWL band of diffuse reflectance filter on the mean percent error of the empirical model for TMRE extraction, the bandwidth is fixed at 10 nm. (b) The corresponding powers [α,β] for the combination of different CWLs.

Figure 8 shows the effect of the CWL of diffuse reflectance filters on the accuracy of the empirical model for TMRE correction when the bandwidth of the diffuse reflectance filters is set to 10 nm. The curves with different colors represent the selection of different CWLs of diffuse reflectance filters (${\mathrm{CWL}}_{\mathrm{ex}}$ or ${\mathrm{CWL}}_{\mathrm{ex}}$). The data in Fig. 8(a) show a minimal effect of diffuse reflectance filters’ CWL on the performance of the empirical model no matter the selection of ${\mathrm{CWL}}_{\mathrm{em}}$ and ${\mathrm{CWL}}_{\mathrm{ex}}$ for diffuse reflectance filters except the case of 570 nm. Fig. 8(b) shows the corresponding powers for each combination of ${\mathrm{CWL}}_{\mathrm{em}}$ and ${\mathrm{CWL}}_{\mathrm{ex}}$. Generally, the absolute power values decrease when the bandwidths increase, the absolute values of each pair of the powers were close to each for all combinations as observed previously.

## 4 Discussion

It is well known that many tumors employ high rates of glycolysis during normoxia.^21^ Beyond the extensively studied “Warburg effect,”^22^ mitochondria have also gained recognition for their distinct contribution to tumor oxidative metabolism.^23^ These metabolic changes can be influenced by the tumor *in vivo* microenvironment, which also plays an important role in the capacity of tumor cells to evade therapies.^24^ Therefore, it is critical to be able to quantify both glycolysis and mitochondrial metabolism *in vivo* to study the tumor metabolism reprograming and its role in tumor survival and recurrence following therapies. Optical spectroscopy has great potential to serve as a cost-effective tool for *in vivo* measures of tumor glycolysis and mitochondrial function. We have developed novel techniques to quantify glucose uptake using glucose analog 2-NBDG^15^ and mitochondrial membrane potential using TMRE^16^ in tumor models *in vivo*.

To enable simultaneous *in vivo* quantification of 2-NBDG and TMRE uptake on small tumor models, we have demonstrated that these two probes are optically compatible when biologically low doses of 2-NBDG and TMRE are used.^15^ We further demonstrated the simultaneous quantification of intrinsic 2-NBDG and TMRE fluorescence along with vascular parameters on solid tumors *in vivo* using a Monte Carlo model-based optical spectroscopy platform.^11^ To further advance our technique toward real-time monitoring of tumor metabolism *in vivo*, we developed an empirical ratiometric method for rapid spectral data processing and demonstrated the technique through tissue-mimicking studies. We showed that the empirical method has the potential to enable real-time intrinsic fluorescence extraction from *in vivo* spectral data due to the inherent simplicity of the proposed approach. In our ratio-metric method, the diffuse reflectance data which contain tissue absorption and scattering information will be used to correct the fluorescence signals. The tissue-mimicking phantoms that we used to evaluate our ratio-metric method have comparable tissue optical properties of mice skin and flank tumors,^20^ thus we believe that our ratio-metric model evaluated by the phantoms will behave similarly for *in vivo* scenario for simultaneous quantification of 2-NBDG and TMRE. In this study, we conducted both 2-NBDG and TMRE phantom studies to demonstrate proof-of-concept of the method due to the general interest in tumor glucose uptake and mitochondrial metabolism for cancer research. We envision that our method will be generally applicable to other metabolic probes such as bodipy to report tumor fatty acid uptake.^25^ However, when a new probe such as bodipy is introduced, additional tests have to be done to ensure the new probe will be optically and biologically compatible with the existing probes.

To implement general use of the empirical model with existing commercial optical filters for spectral data processing, we evaluated the effect of the CWL bands used for correction and their bandwidths on the accuracy of metabolic probe quantification. Generally, we found that the bandwidth had minimal effect on the performance of our technique for 2-NBDG and TMRE extraction when the wavelengths at the probes’ excitation and emission peaks were selected for correction as shown in Figs. 3(a) and 7(a). Based on this, a larger bandwidth of the optical filters would be recommended in the design of an optical system as they typically yield stronger optical signals, which may potentially provide faster data acquisition as minimal integration time may be used. However, we did observe that the selection of CWL bands may significantly affect the performance of the empirical method for intrinsic fluorescence extraction based on the data shown in Figs. 4(a) and 8(a). Proper selection of these wavelength bands will be necessary to enable the high accuracy of the method for spectral data processing.

The system-dependent powers used to correct the distortions can be easily established through one-time tissue-mimicking phantom studies for a given fluorescence probe. As long as the optical properties of an “unknown” sample are within the range of the optical properties of reference tissue-mimicking phantoms, the optimal power values set through these reference phantoms will be adequate to correct the fluorescence measured on the “unknown” sample. To cover a wide range of skin tissue optical properties, the average absorption coefficients were varied from 1.5 to $4.5\;{\mathrm{cm}}^{-1}$ and the average reduced scattering coefficients were varied from 4.5 to $13.5\;{\mathrm{cm}}^{-1}$ based on former independent studies.^20^ The optimal power values reported in this study will be adequate to correct fluorescence measured on skin tissues or flank tumors. However, the power values might be also optical system dependent, thus a new set of reference phantom studies will be always recommended before implementing the method for a new spectroscopy platform.

It is interesting to note that the magnitude of the power values varied dramatically and switch signs for TMRE data but not for 2-NBDG data. This phenomenon is likely due to the fact that the absorption and scattering caused changes in diffuse reflectance within the band for TMRE correction is much smaller than that within the range for 2-NBDG correction as seen in Figs. 1(b) and 5(b). It is possible that these large power values may amplify noise significantly. Using additional terms in the ratio-metric method to scale the signal as a multiplicative constant may potentially avoid potential large changes in the power parameters to improve the robustness of our ratio-metric model. It was also interesting to notice that the absolute values of each pair of the power α and β were close to each for all combinations for both 2-NBDG and TMRE, which opens a potential of reducing two power parameters [α,β] to one single parameter and simplifies the ratiometric model as

$$F_{\mathrm{corr}}(\lambda )=\frac{F_{\text{raw}}(\lambda )}{{\left[R_{\mathrm{ex}}/R_{\mathrm{em}}\right]}^{\alpha }}.$$ (2)

This simplified form of ratiometric model offers the advantage of minimizing the diffuse reflectance calibration errors. We did some simple tests using the simplified form to process our spectral data but achieved slightly lower accuracy, i.e., ∼10% percent error on average. Nevertheless, it is worth considering this simplified version for future data processing as long as a trade-off between the accuracy and system complicity can be made. The simplified model could potentially be adapted to an imaging setup such as a metabolic spectral microscope^15^ for data processing. We envision that the guidelines for selection of wavelength bands and their bandwidths derived from this spectroscopy study will also be applicable to the method adapted for a microscope. However, more rigorous studies will be needed before implementing the method for a microscope.

We conducted tissue-mimicking phantom studies to demonstrate the proof-of-concept of our technique. It will be straightforward to translate the technique for *in vivo* data processing as long as the scaling factors between the phantom data and actual tissue data are found as we reported previously for a Monte Carlo model.^11^^,^^12^ The hemoglobin in the phantoms originated from dehydrated human hemoglobin powder; therefore, oxidization was expected right after phantoms were fabricated in the exposure of air. To stablize the optical properties of phantom set before the spectral measurment, we kept the phantoms in tightly sealed bottle after fabrication and left them overlight before taking the measurement in the following day. The oxidation of hemoglobin in phantoms during this overnight process might be the reason why the absorption peaks of hemoglobin in Figs. 1(b) and 5(b) are still visible but not as prominent as expected. However, this does not impaire the performance of our proposed ratio-metric method for fluorescence correction as confirmed by our independent validation phantom studies. We will further develop and apply the reported empirical method for *in vivo* preclinical data processing in our future study plans. Our phantom study demonstrated that the empirical method can rapidly extract 2-NBDG and TMRE concentrations from distorted optical spectral data with high accuracy. Due to the ratiometric nature of our empirical method for data processing, our technique will potentially facilitate real-time quantification of key metabolic parameters of tumor models *in vivo* using optical spectroscopy techniques, which will advance the translational cancer research with a focus on tumor metabolism reprogramming. Our methodology reported here will also be generally applicable to more metabolic probes and other optical spectroscopy systems, and potentially imaging platforms.

## 5 Conclusion

This work reported an empirical method to quantify the key intrinsic metabolic probes in near real-time at high accuracy. We demonstrated the method for rapid quantification of 2-NBDG and TMRE, two probes reporting glucose uptake and mitochondrial function, from tissue-mimicking phantom studies. The proposed technique will potentially enable real-time *in vivo* monitoring of key metabolic parameters of small tumor models using optical techniques, which will significantly benefit the translational cancer research field.
